# On the study of the transmission networks of blood parasites from SW Spain: diversity of avian haemosporidians in the biting midge *Culicoides circumscriptus* and wild birds

**DOI:** 10.1186/1756-3305-6-208

**Published:** 2013-07-15

**Authors:** Martina Ferraguti, Josué Martínez-de la Puente, Santiago Ruiz, Ramón Soriguer, Jordi Figuerola

**Affiliations:** 1 Departamento de Ecología de Humedales, Estación Biológica de Doñana (EBD-CSIC), Seville E-41092, Spain; 2 Servicio de Control de Mosquitos, Diputación de Huelva, Huelva E-21003, Spain; 3 Departamento de Etología y Conservación de la Biodiversidad, Estación Biológica de Doñana (EBD-CSIC), Seville E-41092, Spain

**Keywords:** Blood parasites, Haemosporidians, *Haemoproteus*, *Plasmodium*, Host parasite interactions, Vector-borne diseases

## Abstract

**Background:**

Blood-sucking flying insects play a key role in the transmission of pathogens of vector-borne diseases. However, at least for the case of avian malaria parasites, the vast majority of studies focus on the interaction between parasites and vertebrate hosts, but there is a lack of information regarding the interaction between the parasites and the insect vectors. Here, we identified the presence of malaria and malaria-like parasite lineages harbored by the potential vector *Culicoides circumscriptus* (Kieffer). Also, we identified some nodes of the transmission network connecting parasite lineages, potential insect vectors and avian hosts by comparing *Haemoproteus* and *Plasmodium* lineages isolated from insects with those infecting wild birds in this and previous studies.

**Methods:**

Using a molecular approach, we analysed the presence of blood parasites in a total of 97 biting midges trapped in the Doñana National Park (SW Spain) and surrounding areas. Also, 123 blood samples from 11 bird species were analyzed for the presence of blood parasite infections. Blood parasites *Haemoproteus* and *Plasmodium* were identified by amplification of a 478 bp fragment of the mitochondrial cytochrome *b* gen.

**Results:**

Thirteen biting midges harboured blood parasites including six *Haemoproteus* and two *Plasmodium* lineages, supporting the potential role of these insects on parasite transmission. Moreover, ten (8.1%) birds carried blood parasites. Seven *Plasmodium* and one *Haemoproteus* lineages were isolated from birds. Overall, six new *Haemoproteus* lineages were described in this study. Also, we identified the transmission networks of some blood parasites. Two *Haemoproteus* lineages, hCIRCUM03 and GAGLA03, were identical to those isolated from *Corvus monedula* in southern Spain and *Garrulus glandarius* in Bulgaria, respectively. Furthermore, the new *Haemoproteus* lineage hCIRCUM05 showed a 99% similarity with a lineage found infecting captive penguins in Japan.

**Conclusions:**

The comparison of the parasite lineages isolated in this study with those previously found infecting birds allowed us to identify some potential nodes in the transmission network of avian blood parasite lineages. These results highlight the complexity of the transmission networks of blood parasites in the wild that may involve a high diversity of susceptible birds and insect vectors.

## Background

Vector-borne diseases constitute a major problem affecting human and animal populations [1]. The Haemosporidians (Phylum Apicomplexa) is a group of vector-borne parasites requiring both vertebrate and insect hosts to complete their life cycle. Avian haemosporidians are the largest group of haemosporidians by species number, which include, among others, the malaria parasite *Plasmodium* and the closely related genera *Haemoproteus* and *Leucocytozoon*[2,3]. These parasites require the intervention of blood-sucking insects (Diptera) during their sexual and sporogonic phases along with an intermediate vertebrate host for the merogony phase and the development of gametocytes [3]. *Plasmodium* and *Haemoproteus* parasites can infect a broad range of insects showing, however, a certain degree of specificity. Avian *Plasmodium* species are transmitted by blood-sucking mosquitoes (Culicidae), while *Haemoproteus* are transmitted by biting midges (Ceratopogonidae) and louse flies (Hippoboscidae) [1,4].

Nowadays, the vast majority of avian malaria studies focus on the interaction between blood parasites and vertebrate hosts [5-8]. However, considering the large number of studies on avian haemosporidians, there is a lack of information targeting the parasites infecting insect vectors (for a recent review [9]). The use of molecular techniques has significantly improved the capacity to identify the networks of avian blood-parasite transmission and has led to a new era on the research of parasite–insect interactions. However, despite the recent increase in interest toward studies on insects as major vectors in the transmission of avian malaria parasites, the role of vectors on population dynamics of avian malaria parasites in natural ecosystems has been poorly studied. In addition, most studies on potential vectors have been focused on particular insect groups, mainly on mosquitoes [10-14] and black flies [15-17]. In contrast, other groups, as is the case of the biting midges *Culicoides* Latreille (Diptera: Ceratopogonidae) have been comparatively poorly studied.

Biting midges *Culicoides* are a diverse and widespread genus with more than 1400 species in the world [18], with at least 81 of them present in Spain [19]. Biting midges transmit pathogens with sanitary importance, including the Bluetongue virus and the emergent Schmallenberg virus [18,20], in addition to the majority of avian *Haemoproteus* (subgenus *Parahaemoproteus*) parasites. To fully understand the role of biting midges in the transmission network of blood parasites is essential to reveal the interactions between insects, avian hosts and the blood parasites harboured by the insects. Different studies have revealed the avian hosts of biting midges by identifying the blood meal origin [21,22] or the insect species attracted to birds [23-25]. However, to our knowledge, only three molecular studies have screened the blood parasites harbored by biting midges. In one study, authors identified the blood parasite lineages from individual whole parous females [24] while, in another, *Haemoproteus* lineages were isolated from pools containing the head-thorax of blood-fed females [17]. Additionally, Santiago-Alarcon *et al*. [21] isolated blood parasite lineages from the abdomen of blood-fed *Culicoides*. Therefore, further studies on the role of *Culicoides* species on the transmission of blood parasites are necessary, as this information is currently missing for the vast majority of *Haemoproteus* species. This is especially relevant in light of the fact that that these parasites play a key role on the health status and survival probability of avian species [26-28].

Here, using a molecular approach, we identified: i) the *Haemoproteus* and *Plasmodium* lineages harboured by the biting midge *Culicoides circumscriptus* (Kieffer) captured in different areas around the Doñana National Park and ii) the *Haemoproteus* and *Plasmodium* lineages infecting wild birds in the studied area in order to identify some nodes of the transmission networks connecting parasite lineages, potential insect vectors and avian hosts. We focus our study on the biting midge *C*. *circumscriptus* because this species: i) has an ornithophilic behaviour [22,23,25], ii) has a broad distribution covering most of Europe and North Africa being considered the most abundant ornithophilic species in southern Spain [29] and iii) harbours different blood parasite lineages [24]. Altogether, these studies suggest that *C*. *circumscriptus* may play a key role on avian blood parasite transmission in the wild.

## Methods

### Study area and sample collection

**Figure 1:**
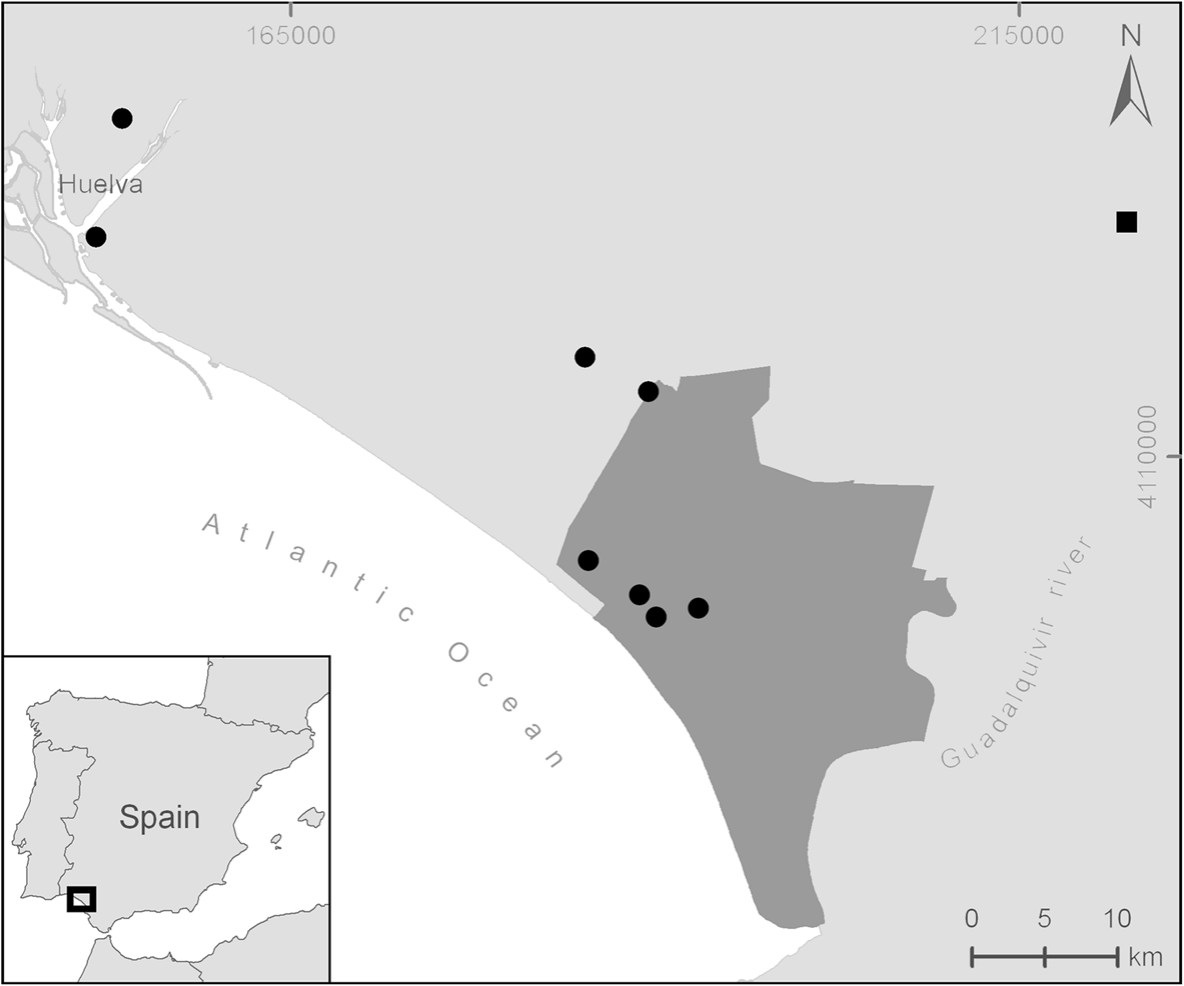
Distribution of the insect (circles and square) and bird (square) sampling sites in the Doñana National Park (in grey) and surrounding areas.

We studied *Culicoides* specimens captured from April to June 2008 in different localities from the Doñana National Park and surroundings areas (SW Europe: 38° 42′–35° 59′ N; 7° 30′–1° 38′ W) (Figure 1). Insect trapping was conducted during 24 hours using Center for Disease Control (CDC) incandescent light-traps, powered by a 6V battery and baited with CO_2_. The sampling intervals were two consecutive days per week or two weeks. Traps were hung at 1.60 meters above ground level with the exception of two localities that were placed at two different heights, of 1.60 and 15 meters above ground level. This was done in order to maximize captures of the ornithophilic *C*. *circumscriptus*, a species that prefer the canopy level [30]. Insect catches were stored *in situ* in dry ice and maintained frozen until identification and processing in the laboratory.

As a part of a long-term study on the transmission of West Nile Virus (see [31]), we randomly selected blood samples from 123 birds belonging to 11 species (Table 1) captured from January to December 2009 in the Cañada de los Pájaros (Seville, Spain; 6°14′W, 36°57′N), one of the insect trapping localities. Birds were captured without damage using walk-in-traps and released at the same place after sampling. Blood samples were taken with syringes from the tarsal vein and each bird was marked with numbered aluminium rings and released after manipulation. The volume of blood extracted (up to 1 ml) depended on the size of the species and never exceeded 1% of avian body mass. In the same day, blood samples were centrifuged and the cell fraction was used for blood parasite identification. Insect collection and bird sampling were performed with all the necessary permits from landowners and regional Department of the Environment (Consejería de Medio Ambiente, Junta de Andalucía).

**Table 1:** Number of birds sampled and number of infected individuals (in brackets) for each avian species studied

**Scientific name**	**Common name**	**Individuals (infected)**	***Plasmodium *lineage**	***Haemoproteus *lineage**
*Anas acuta*	Northern pintail	8 (1)	Rinshi-1	
*Anas strepera*	Gadwall	7 (0)		
*Aythya ferina*	Common pochard	10 (0)		
*Bubulcus ibis*	Cattle egret	13 (3)	GAL-2012+Rinshi-1(*)	hBUBIBI01
			P15	
*Ciconia ciconia*	White stork	15 (1)	Rinshi-1	
*Egretta garzetta*	Little egret	10 (1)	Delurb5	
*Fulica cristata*	Crested coot	24 (1)	pSPHUjJ	
*Gallinula chloropus*	Common moorhen	15 (1)	AFTRU5	
*Himantopus himantopus*	Black-winged stilt	6 (0)		
*Marmaronetta angustirostris*	Marbled duck	10 (1)	Rinshi-1	
*Tadorna tadorna*	Common shelduck	5 (1)	Donana10	

The information on the blood parasite lineages identified for each avian species is given in different columns for *Plasmodium* and *Haemoproteus*.

(*) One *Bubulcus ibis* showed co-infection by two different lineages.

### Insect identification

All biting midges were transferred to vials containing 70% ethanol and sorted according to their feeding status under a Zeiss Discovery V8 stereomicroscope. Parous *C*. *circumscriptus* females, those that have fed on a host at least once in their life but that did not have visual evidence of blood remains in the abdomen, were isolated as they may be susceptible to transmitting blood parasites. They were identified to species level based on their wing pattern using appropriate taxonomic keys [32,33] and the parous status based on the presence of burgundy-red pigmented abdomen that develops during the 1st gonotrophic cycle [34]. Some males and nulliparus females were dissected and mounted for more accurate morphological diagnosis.

### DNA extraction and blood parasite identification

Genomic DNA from 97 randomly selected *Culicoides* was individually extracted using the DNeasy Blood and Tissue kit (QIAGEN, Hilden). For blood samples, DNA was isolated using the following procedure: a portion of blood clot was pipetted into vials containing 300 μl of SET buffer (100 mM NaCl, 50 mM Tris–HCl, pH 8.50 mM EDTA, pH 8.0, SDS 1%), 5 μl proteinase K (20 mg/ml) and 10 μl DDT (1 M), and subsequently maintained overnight in a incubating shaker at 55°C. Once the digestion was completed, an equal volume (300 μl) of 5 M LiCl was added to each tube and each sample was mixed thoroughly by inversion with the addition of 630 μl of chloroform-isoamyl alcohol (24:1). Samples were spun and the supernatant (around 500 μl) was carefully transferred to a new tube and the DNA precipitated with absolute ethanol. After recovery by centrifugation, the DNA was dried and washed with 70% ethanol and the final pellet was recovered and stored in water [35].

Blood parasites *Haemoproteus* and *Plasmodium* were identified using the protocol detailed by Hellgren *et al*. [7] based on the amplification of a 478 bp fragment (excluding PCR primers) of the mitochondrial cytochrome *b* gen. This procedure is based on a first PCR using primers HaemNFI (5′CATATATTAAGAGAAITATGGAG3′) and HaemNR3 (5′ATAGAAAGATAAGAAATACCATTC3′), followed by a nested PCR to amplify *Haemoproteus* and *Plasmodium* genera using the primer pair HaemNF (5′ATGGTGCTTTCGATATATGCATG3′) and HaemNR2 (5′GCATTATCTGGATGTGATAATGGT3′).

Parasite determination was conducted at least twice per sample to avoid false negative samples [36]. Both negative controls for PCR reactions (at least one per plate) and DNA extraction were included in the analysis. Due to strict laboratory protocols and no evidence of contamination in any negative controls, we have assumed that false positives were negligible. Lineages were identified by sequencing the amplicons using the Big Dye 1.1 technology (Applied Biosystems). Labelled DNA fragments of positive PCR products were resolved with an ABI 3130xl automated sequencer (Applied Biosystems) using the same forward and reverse primers used in the nested-PCR amplification. Sequences were edited using the software Sequencher™ v 4.9 (Gene Codes Corp., © 1991–2009, Ann Arbor, MI 48108) and identified by comparison with the GenBank DNA sequence database (National Center for Biotechnology Information) to assign unknown cytochrome *b* sequences to previously identified parasite lineages.

## Results

### Blood parasite identification from biting midges *Culicoides*

Thirteen of 97 parous *Culicoides* females harboured blood parasites. A similar prevalence was found in traps at 1.60 (9 of 62; 14.5%) and at 15 (4 of 35; 11.4%) meters above the ground level (χ^2^=0.01, 1 df, p=0.91). Six *Haemoproteus* and two *Plasmodium* different lineages were isolated. One *Haemoproteus* lineage had a 100% overlap with lineage GAGLA03 (GenBank accession number: GU085197), isolated in Bulgaria from *Garrulus glandarius* and five new *Haemoproteus* lineages were described in this study and sequences deposited in GenBank (number of positive midges and accession numbers are shown in brackets): hCIRCUM01 (from one biting midge, KC994896), hCIRCUM02 (from one biting midge, KC994897), hCIRCUM03 (from three biting midges, KC994898), hCIRCUM04 (from two biting midges, KC994899) and hCIRCUM05 (from one biting midge, KC994900). In these isolates, hCIRCUM03 was identical to a lineage isolated from *Corvus monedula* from southern Spain in our laboratory (authors unpublished data) and hCIRCUM05 showed a 99% similarity with the *Haemoproteus* lineage hAPPAjS (Genbank accession number: AB604312) isolated in Japan from an *Aptenodytes patagonicus* captive in a zoo. Moreover, the two *Plasmodium* lineages isolated were identical to previously described sequences corresponding to Rinshi-1 (from *Plasmodium relictum*) and pSPHUjJ isolated from one and two biting midges, respectively. The rest of the lineages showed a similarity ≤ 98% with sequences deposited in GenBank database. Sequences obtained from a single biting midge showed double peaks suggesting co-amplification of at least two different lineages.

### Blood parasites from birds

Seven *Plasmodium* and one *Haemoproteus* lineages were isolated from 10 infected birds (Table 1). One *Bubulcus ibis* showed mixed infections by two *Plasmodium* lineages (GAL-2012 and Rinshi-1). Moreover, seven *Plasmodium* lineages were isolated and completely matched to previously described lineages (sequences published in GenBank database): AFTRU5, GAL-2012 (=GRW6 from *Plasmodium elongatum*), Delurb5, Donana10, P15, pSPHUjJ and Rinshi-1. In addition, hBUBIBI01, a new *Haemoproteus* lineage was isolated from a *Bubulcus ibis* and the sequence deposited in GenBank (accession number: KC994901).

## Discussion

Here, we studied the lineage diversity of blood parasites harbored by the potential vector *C*. *circumscriptus* and those parasites infecting different bird species from the same area. A total of seven *Haemoproteus* and seven different *Plasmodium* lineages were isolated from biting midges and birds. Although studies on blood parasites infecting both birds and potential insect vectors are increasing, information on the potential associations between avian malaria parasites and vector species are still scarce and further studies are crucial to fully understand the transmission networks of these parasites in the wild [15,24,37,38]. Here, we present valuable information in this respect, but we identify the potential vector and vertebrate hosts of only a few parasite lineages. These results highlight the difficulties in identifying both vertebrate hosts and potential vectors of blood parasite lineages. Based on the previously identified vertebrate hosts of blood parasite lineages isolated from biting midges in this study, we could conclude that *C*. *circumscriptus* may be involved in the transmission of *Haemoproteus* parasites from *Garrulus glandarius* and *Corvus monedula*, both species belonging to the order Passeriformes. In this respect, it could be possible that we failed to identify more vertebrate hosts of blood parasites because we sampled bird species other than passeriforms. Also, *C*. *circumscriptus* could play a significant role in the dynamics of blood parasite transmission infecting exotic species keep in captivity, as may be the case of the penguin *Aptenodytes patagonicus* in Japan. The last result should be considered important under a conservation perspective as infection by blood parasites *Haemoproteus* could have dramatic consequences on captive exotic species in zoos [26,39], or in species that do not usually enter into contact with the vector and the pathogen [40].

### Haemosporidian parasites in biting midges

The prevalence of infection by blood parasites found in parous biting midges in this study was similar to those values reported for *C*. *circumscriptus* in central Spain [24]. Contrary to the case of another potential vector of avian blood parasites i.e. mosquitoes, it is possible to visually assign parous biting midge *Culicoides* females according to the pigmentation of their abdomen [34]. This fact may allow researchers to test for the presence of blood parasites only in those females that previously fed on a vertebrate host. Thus, the economic cost of testing unfed females could potentially be reduced, increasing the reliability of prevalence estimates by removing the bias introduced by differences in parous rates among populations, seasons, or species. Also, this fact may increase the parasite prevalence estimation with respect to those studies including unfed insects, as may be the norm on studies on mosquitoes (e.g. [41,42]). However, in some *Culicoides* species, a percentage of newly emerged females without previous contact with vertebrate hosts, may present abdominal pigmentation being potentially incorrectly identified as parous females [43]. However, as far as we know, this is not the case of *C*. *circumscriptus*.

In addition to the *Haemoproteus* lineages, we isolated two *Plasmodium* lineages from three parous *C*. *circumscriptus* biting midges. Avian *Plasmodium* species are transmitted by mosquitoes most of them belonging to the *Culex* genus [3]. Despite this, nowadays, some studies have isolated *Plasmodium* lineages from biting midges [9,24]. However, these studies were based exclusively on molecular analysis and PCR identification of a parasite lineage does not necessary imply vector competence [44], as positive amplifications could be representative of DNA amplifications of abortive parasite development [45].

### Haemosporidian parasites in birds

Although our results on birds were based on a relatively low sample size, we isolated a considerably high diversity of blood parasite lineages. The most common parasite lineage isolated from birds was Rinshi-1 corresponding to *P*. *relictum*, which may be potentially transmitted by several mosquito species in the studied area [42]. Also, we have isolated the lineage GAL-2012 (=GRW6) from birds, which have been assigned to the morphospecies *Plasmodium elongatum*[46]. Both *P*. *relictum* and *P*.*elongatum* are known to have a broad distribution infecting a wide range of avian species [3,46]. Interestingly, a genetic lineage from *P*. *elongatum*, the parasite species here isolated from *Bubulcus ibis* was previously found infecting the related *Ardea herodias* in the USA [see [46]. Only a single *Haemoproteus* lineage was isolated from birds in this study. This could be due to the lack of samples from Passeriformes and, at least in part, to the fact that birds were sampled in a locality a relatively long way from most of the other insect trapping localities.

Moreover, although the prevalence is low, we isolated blood parasites from the endangered *Fulica cristata* and *Marmaronetta angustirostris*. Further studies are necessary in order to identify the potential impact of blood parasite infection on the health and survival of these species.

## Conclusions

The biting midge *C*. *circumscriptus* may play a key role in some transmission networks of different genetic lineages of the malaria-like parasite *Haemoproteus*. Overall, results from this study highlight the difficulties in identifying the nodes of these complex transmission networks of blood parasites in the wild that may involve a high diversity of susceptible hosts and insect vectors.

## Competing interests

The authors declare that they have no competing interests.

## Authors’ contributions

MF, JMP, SR, RS, JF were involved in development of the study design. MF and JMP performed the laboratory analysis and data analysis. SR, RS, JF contributed samples/reagents/materials/analysis tools. All authors have read and approved the manuscript.
